# Removal of Iron and Copper Ions and Phenol from Liquid Phase by Membrane Based on Carbonaceous Materials

**DOI:** 10.3390/ma17122788

**Published:** 2024-06-07

**Authors:** Joanna Przybyl, Aleksandra Bazan-Wozniak, Faustyna Poznan, Agnieszka Nosal-Wiercińska, Judyta Cielecka-Piontek, Robert Pietrzak

**Affiliations:** 1 Department of Applied Chemistry, Faculty of Chemistry, Adam Mickiewicz University in Poznań, Uniwersytetu Poznańskiego 8, 61-614 Poznań, Poland; asia.krason@amu.edu.pl (J.P.); aleksandra.bazan@amu.edu.pl (A.B.-W.); faupoz@st.amu.edu.pl (F.P.); 2 Department of Analytical Chemistry, Institute of Chemical Sciences, Faculty of Chemistry, Maria Curie-Skłodowska University in Lublin, Maria Curie-Skłodowska Sq., 3, 20-031 Lublin, Poland; agnieszka.nosal-wiercinska@mail.umcs.pl; 3 Department of Pharmacognosy, Faculty of Pharmacy, Poznan University of Medical Sciences, Rokietnicka 3, 60-806 Poznan, Poland; jpiontek@ump.edu.pl

**Keywords:** cellulose acetate membrane, physicochemical properties, carbonaceous adsorbents, microwave oven, organic and inorganic pollutants

## Abstract

The present work reports an effective method for the removal of inorganic and organic pollutants using membranes based on different carbonaceous materials. The membranes were prepared based on cellulose acetate (18 wt. %), polyvinylpyrrolidone as a pore-generating agent (2 wt. %) and activated carbon (1 wt. %). Activated carbons were developed from residues after extraction of the mushroom *Inonotus obliguus* using microwave radiation. It has been demonstrated that the addition of activated carbon to the membranes resulted in alterations to their physical properties, including porosity, equilibrium water content and permeability. Furthermore, the chemical properties of the membranes were also affected, with changes observed in the content of the surface oxygen group. The addition of carbon material had a positive effect on the removal of copper ions from their aqueous solutions by the cellulose–carbon composites obtained. Moreover, the membranes proved to be more effective in the removal of copper ions than iron ones and phenol. The membranes were found to show higher effectiveness in copper removal from a solution of the initial concentration of 800 mg/L. The most efficient in copper ions removal was the membrane containing urea-enriched activated carbon.

## 1. Introduction

In recent years, the study of the effects of pollution on the environment and human health has received increasing attention. The negative effects of heavy metals and phenol in water on both the human body and the environment have been known for years. Consumption of water with increased concentrations of iron and copper ions causes health problems such as disorders of the nervous and cardiovascular systems, abnormal metabolism or cancer [1,2]. Conversely, phenol, an organic aromatic compound, has been demonstrated to exert a detrimental impact on the functioning of the human body, manifesting as skin irritation, cardiac, renal and hepatic impairment, visual impairment and metabolic dysfunction [3]. Furthermore, elevated phenol content and iron and copper ions in the hydrosphere have a negative effect on aquatic organisms, causing, among other things, oxidative stress [4]. The above facts serve to confirm the validity of developing new and modifying existing methods to effectively remove these pollutants from both ground and surface waters [5,6].

Membrane processes represent one of the most popular methods for contaminant removal in chemical engineering [7,8]. At the synthesis stage, membranes can be modified by the addition of a pore-generating agent (e.g., polyvinylpyrrolidone) or materials (e.g., activated carbons) to improve the removal efficiency [9,10]. Modified membranes are obtained using the phase inversion method, the most popular of the flat sheet membrane fabrication methods. This method involves the formation of a mixture comprising polymer, blowing agent, additional components and solvent, which is then spread on a substrate. After the solvent has evaporated, the mixture is immersed in a coagulation bath, such as deionised water [11,12].

At the stage of membrane synthesis, it is possible to modify the membranes with, for example, amines, metal oxides or activated carbons. This is discussed in detail in references [13,14]. Activated carbons are frequently employed as adsorbents for contaminants, both in the liquid and gaseous phases [15]. Due to their large specific surface area and microporous structure, these materials are effective adsorbents of compounds such as dyes, metal ions or organic compounds [16,17]. 

This paper presents the results of a study on the preparation, characterisation and efficiency in the removal of phenol and iron and copper ions from the liquid phase of membrane composites obtained on the basis of cellulose acetate and carbonaceous materials. The membranes were modified with the blowing agent polyvinylpyrrolidone. In order to test the influence of the type of carbonaceous material on the properties of the resulting cellulose–carbon composite, carbonaceous material was added during preparation. Tests were conducted on both pure membranes and membranes that had been modified with char, activated carbon derived from it and nitrogen-modified activated carbon. Compared to our previous work, the novel aspect of this research is the use of residues from the extraction of the fruiting body of the chaga mushroom (*Inonotus obliguus*). This precursor is a plentiful waste product with no discernible use. Furthermore, this study introduces a novel approach to synthesising membranes based on carbon materials, which enables the production of cost-effective adsorbents. This strategy also enhances environmental sustainability and provides a foundation for further research in the field of sustainability.

## 2. Materials and Methods

### 2.1. Materials

Cellulose acetate (acetyl content 39.8%, CA) was procured from Sigma Aldrich (Darmstadt, Germany) and employed as a membrane material. N,N-dimethylformamide (˃99.8%, DMF) was sourced from Avantor Performance Materials Poland S.A. (Gliwice, Poland) and utilized as a solvent. Polyvinylpyrrolidone (˃99%, PVP, 10,000 g/mol) as a pore former was provided by Sigma Aldrich (Sigma–Aldrich, Darmstadt, Germany). The carbonaceous materials added to the membranes were char (C), activated carbon (AC) and nitrogen-enriched activated carbon (NAC). The precursor carbon material was derived from the residue resulting from the methanol extraction of the fruiting body of the chaga mushroom (*Inonotus obliquus*). The fruiting body was sourced from a birch trunk. Following drying, the fruiting body was ground into a fine powder. Then, methanol removal was conducted. For this purpose, 2.50 g of powder was weighed and extracted into a reflux cooling bath at 80 °C. Carbon C was obtained by carbonisation of the precursor in a nitrogen atmosphere (150 mL/min) at 500 °C for 60 min (technical nitrogen 4.0, Linde Gaz Poland). Activated carbon (AC) was obtained by physical activation of the precursor with carbon dioxide (250 mL/min) at 800 °C for 30 min (technical CO_2_ 2.8, Linde Gaz Poland). Nitrogen-enriched activated carbon (NAC) was prepared by mixing 40% urea (Chempur, Piekary Śląskie, Poland) with char (C) in a mass ratio of 1:1, followed by activation with CO_2_ (250 mL/min) for 15 min at 500 °C. Carbonisation and activation processes were carried out in a microwave oven (Phoenix, CEM Corporation, Matthews, IL, USA).

### 2.2. Preparation of CA Membranes

Casting solutions of CA 18 wt. %, 2 wt. % of PVP and 1 wt. % of activated carbon were prepared by mixing the ingredients in a flask. The resulting casting solution was then left to rest for approximately 24 h to allow for the complete release of bubbles. Subsequently, the casting solution was cast onto a glass plate using a stainless-steel knife to create a casting film with a thickness of 300 µm. The film was then exposed to the atmosphere for 40 s and subsequently immersed in a coagulation bath of deionised water. The as-prepared cast solution films were immersed and maintained in a deionised water bath at 25 °C for 24 h, after which the membranes formed were washed for a further 24 h using deionised water. The membranes obtained were designated as M, MC, MAC and MNAC.

### 2.3. Membrane Structure Characterisation

#### 2.3.1. Porosity and Equilibrium Water Content

The membrane porosity was determined by the mass loss of wet membrane after drying. The membrane sample was mopped with water on the surface and weighed under wet status. Then, the membrane sample was dried until a constant mass was obtained. The membrane porosity [18] ε was evaluated from the following equation:

(1) $$\varepsilon =\frac{W_{w}-W_{d}}{\rho \times V}\times 100\%$$

where W_w_ is the mass of a wet membrane sample (g), W_d_ is the mass of dry state membrane sample (g), ρ pure water density (g/dm^3^) and V- is the volume of a membrane in wet state (L).

The equilibrium water content [19] (EWC) was determined by Equation (2):

(2) $$EWC=\frac{W_{w}-W_{d}}{W_{w}}\times 100\%$$

Two parallel series of measurements were conducted, and the resulting data were presented as the arithmetic mean of the values obtained in each series.

#### 2.3.2. Contact Angle

The contact angle between water and the membrane was directly measured using a contact angle measuring instrument (G10, KRUSS, Halle, Germany). In order to evaluate the membrane’s hydrophilicity, deionised water was used as a probe liquid in all measurements. In order to minimise the experimental error, the contact angle was measured at five random locations for each sample, and the average was reported.

#### 2.3.3. Surface Oxygen Groups

The surface properties were characterised using potentiometric titration experiments conducted with the aid of the 809 Titrando equipment manufactured by Metrohm (Opacz-Kolonia, Polnad). The instrument was configured to collect equilibrium pH data. The materials were weighed and placed in a container at a temperature of 25 °C. The container was then filled with 50 mL of 0.01 M NaNO_3_ and allowed to equilibrate overnight with the electrolyte solution. To eliminate the influence of atmospheric CO_2_, the suspension was continuously saturated with N_2_. The carbon suspension was continuously stirred throughout the course of the measurements. The volumetric standards NaOH (0.1 M) or HCl (0.1 M) were employed as titrants in accordance with the methodology described in reference [20]. For each sample, two parallel determinations were conducted.

#### 2.3.4. Membrane Performance Characterisation

The water permeability of the membranes was quantified in a stainless-steel cell, with an effective membrane area of 19.6 cm^2^. Prior to testing, the membranes were subjected to deionised water at 3 bar for approximately 1.5 h. The pure water flux was then measured at 3 bar, 23 ± 1 °C and a crossflow velocity of 0.22 m/s. The pure water flux was calculated using the following equation:

(3) $$J_{w}=\frac{V}{A\times \Delta t}$$

where J_w_ (L/(m^2^×h)) is the pure water flux, V (L) is the volume of permeated water, A (m^2^) is the effective membrane area and Δt (h) is the permeation time.

The experiments were conducted using compressed nitrogen gas and three types of solutions: phenol, copper and iron ions. The initial concentrations of the solutions were varied (15 and 25 mg/L for phenol, 800 and 1000 mg/L for copper ions, 12 and 20 mg/L for iron ions). The selection of concentrations is informed by previous research [19,21]. All measurements were conducted at 3 bar in triplicate. The final concentrations of the solutions were analysed using a double-beam UV–VIS spectrophotometer (Agilent, Santa Clara, CA, USA) at 506 nm for phenol [22], 620 nm for copper ions and 487 nm for iron ions. The rejection of this compound was calculated using Equation (4):

(4) $$R=\left(1-\frac{C_{p}}{C_{f}}\right)\times 100\%$$

where C_p_ and C_f_ (mg/mL) were phenol, copper and iron concentrations in the permeate and the feed solutions, respectively.

The membrane resistance was evaluated in accordance with Darcy’s law [22] by calculating the resistance in the series of models as follows (5):

(5) $$J=\frac{\Delta P}{{\mu R}_{t}}$$

where J (L/(m^2^×h)) is the permeate flux, ΔP is the transmembrane pressure (TMP), µ is the dynamic viscosity of permeate (Pa × s) and R_t_ is the total filtration resistance (m/s). The resistance in the series of models combines various resistances causing flux decline as follows (6):

(6) $$R_{t}=R_{m}+R_{p}+R_{c}$$

The total filtration resistance, designated as R_t_, is the sum of various resistances, including that of the membrane itself, R_m_, pore-blocking resistance, R_p_, and cake resistance, R_c_. The intrinsic membrane resistance (R_m_) can be estimated from the initial pure water flux (3). The fouling resistance (R_p_) is a consequence of pore plugging and the irreversible adsorption of foulants on the membrane pore wall or surface. The cake resistance (R_c_) induced by the formation of a cake layer on the membrane surface was calculated from the water flux after pure water washing [23,24].

The behaviour of the detail membrane in the context of fouling was studied in accordance with the following methodology. Initially, the pure water flux of the membrane J_w1_ (L/(m^2^×h)) was evaluated at 3 bar. Subsequently, an aqueous solution of phenol (15 and 25 mg/L), copper ions (800 and 1000 mg/L) and iron ions (12 and 20 mg/L) was introduced into the ultrafiltration system. Following a filtration period of 30 min, the membrane was flushed with pure water for a further 10 min, after which the pure water flux of the membrane, designated J_w2_ (L/(m^2^×h)), was measured. Two parallel series of measurements were conducted, and the resulting data were presented as the arithmetic mean of the values obtained in each series. The flux recovery ratio (FRR) was calculated using Equation (7) in order to evaluate the antifouling properties of the membrane.

(7) $$FRR=\frac{J_{w2}}{J_{tw1}}\times 100\%$$

### 2.4. Carbonaceous Materials Characterisation

The Thermo Scientific FLASH 2000 Elemental Analyzer was utilized for analysing the elemental composition (C, N, H and S) of all carbon materials. The elemental oxygen content was determined by the difference method. Ash content was assessed by subjecting the samples to combustion in a microwave muffle furnace (Phoenix model, CEM Corporation, Matthews, IL, USA) at 815 °C for 60 min.

The prepared carbon materials underwent characterisation for their porous properties utilizing nitrogen adsorption/desorption isotherms measured at 77 K using an AutosorbiQ instrument, provided by Quantachrome Instruments (Boynton Beach, FL, USA). The detailed procedure for carrying out the measurements was previously presented in our article [17].

The SEM images of biocarbons were captured using the scanning electron microscope Quanta 3D FEG (FEI, Field Electron and Ion Co., Hillsboro, OR, USA). pH values and the content of oxygen functional groups of adsorbents were determined following the procedure outlined in our earlier article [25]. Additionally, iodine adsorption analysis was conducted for the obtained carbon materials [25].

## 3. Results

### 3.1. Chracterisastion of the Carbon Materials

Table 1 presents the results of elemental analysis for the carbon materials obtained. A review of the data presented in Table 1 reveals that sample C exhibits the lowest elemental carbon content, which also results in the highest oxygen content for the same material. In contrast, the percentages of hydrogen and nitrogen were 2.1 wt. % and 2.9 wt. %, respectively. Further analysis of the data presented in Table 1 revealed that the activation of the precursor with carbon dioxide and the impregnation of sample C with urea resulted in a notable alteration of the carbon materials obtained. The NAC sample was characterised by its highest concentration of elemental carbon (86.9 wt. %). The carbon obtained by direct activation of the starting material with carbon dioxide exhibited a carbon content that was over 10 wt. % lower than that of NAC activated carbon. In the case of AC and NAC samples, an increase in the C^daf^ content was accompanied by an increase in the proportion of hydrogen and oxygen. Modification with urea allowed obtaining an adsorbent containing in its structure N^daf^—4.7 wt. %. Further analysis of the data revealed that the AC and NAC carbonaceous materials exhibited a low sulphur content, with a maximum of 0.1 wt. %. In contrast, the oxygen content of these samples was found to be between 4.8 wt. % and 5.3 wt. %. It is also pertinent to mention that each of the carbon materials obtained exhibits a markedly high ash content. The mineral substance in question has a weight percentage range of 7.8 wt. % to 12.1 wt. %. The high ash content of the obtained coal materials suggests that ash may be present within the pores of the carbon structure.

The results of the textural studies (Table 2) indicate that the thermochemical treatment of the residue following the extraction of the fungus *Inonotus obliquus* did not facilitate the development of a porous structure effectively. This is demonstrated by the specific surface areas of the obtained activated carbons, which are 125 (sample C), 749 (sample AC) and 888 m^2^/g (sample NAC), respectively. The most developed surface area was characterised by NAC adsorbent, which is the only one with a specific surface area greater than 400 m^2^/g.

**Figure 1:**
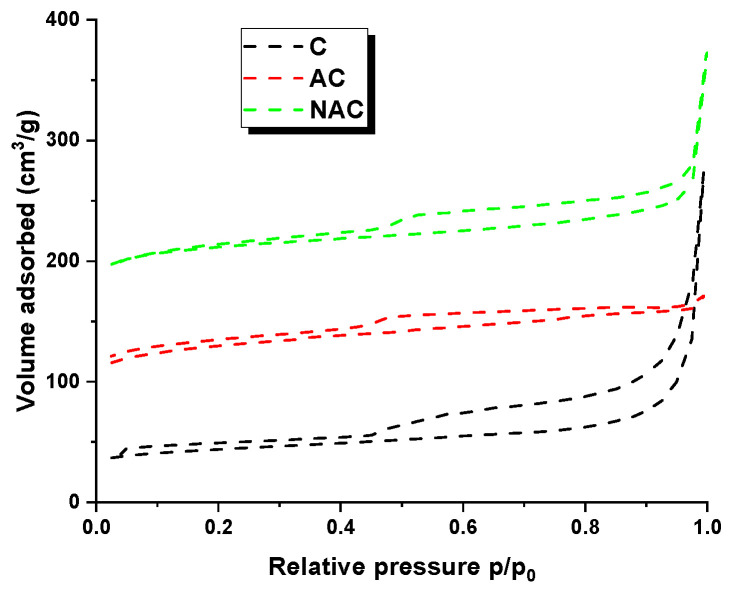
Low-temperature nitrogen adsorption/desorption isotherms for carbon materials obtained.

The data presented in Table 2 indicate a correlation between the specific surface area and the iodine numbers obtained for the carbon adsorbents produced. The tested adsorbents exhibited a porous structure comprising small mesopores, as evidenced by the average pore diameter values, which ranged from 4.15 to 7.32 nm. Furthermore, the nitrogen desorption adsorption isotherms, as illustrated in Figure 1, demonstrated the presence of such pores. In accordance with the IUPAC classification, the isotherms depicted in Figure 1 are indicative of type IV. A defining characteristic of the type IV isotherm is the presence of a clearly delineated hysteresis loop, which is associated with capillary condensation in the region of mesopores. The H4-type hysteresis loops visible in the isotherms around the p/p_0_ pressure of approximately 0.4 suggest the condensation of nitrogen in the mesopores. This indicates that the tested coals have developed mesoporosity [26].

**Figure 2:**
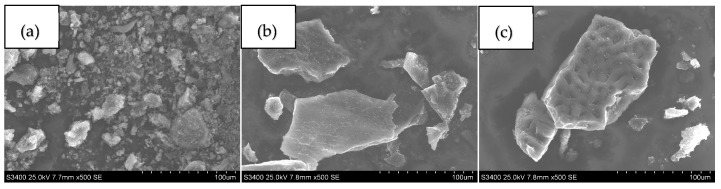
SEM images of the carbon adsorbents obtained: (**a**) sample C, (**b**) sample AC and (**c**) sample NAC.

SEM images of the adsorbent samples are presented in Figure 2. Regarding char samples, the ash content may account for the brighter fragments observed.

**Figure 3:**
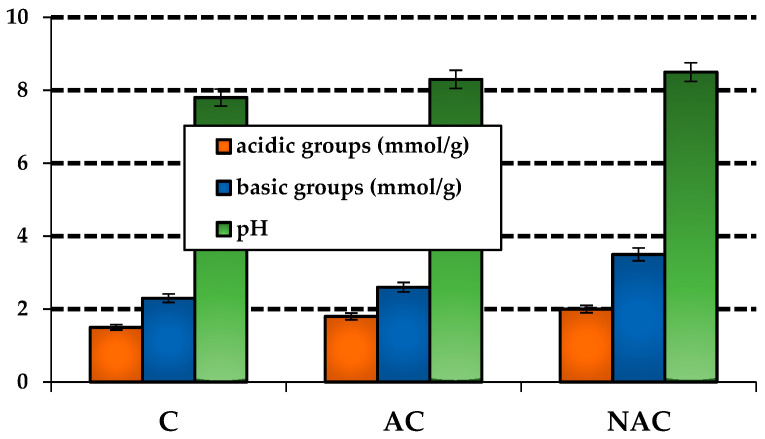
Acid–base properties of the carbon adsorbents.

The data presented in Figure 3 indicate that the obtained adsorbents exhibit comparable acid–base properties. As evidenced by the presented data, the type and quantity of surface oxygen groups are contingent upon the variant of carbon sample production. Regardless of the preparation variant, all coals exhibited acidic and basic groups on their surface. The NAC sample exhibited the highest proportion of both groups. The carbon in question exhibited 2.0 mmol/g of acidic functional groups and 3.5 mmol/g of basic groups on its surface. Furthermore, the data revealed a prevalence of basic groups over acidic ones for all samples. The predominance of basic functional groups is also corroborated by the pH values presented in Figure 3, which range from 7.8 to 8.5.

### 3.2. Physicochemical Properties and Adsorption Characteristics of the Membranes

Table 3 presents the results for the structural parameters and wetting angle values of the materials under investigation. The data in Table 3 indicate that the char-enriched membrane exhibited the highest values for porosity (68.60%) and equilibrium water content (88.21%). The values for the MNAC membrane and the membrane without added char material were slightly lower. The lowest porosity and equilibrium water content values were determined for the MAC material. The values of the EWC parameter indicate a high susceptibility of the materials tested to drying out, which is a significant factor to be considered when determining the most appropriate storage environment. The analysis of the wetting angles revealed that the MAC material exhibited the most hydrophilic surface character, while the membrane without added carbon material exhibited the least hydrophilic character.

Table 4 shows the content of acid and basic oxygen groups on the surface of the extracted materials.

From the values obtained, it can be seen that the MAC material had the highest number of acid functional groups (5.19 mmol/g). On the surface of the pure membrane, the number of acid groups was 3.69 mmol/g. For the other two membranes, however, the values were much lower at 2.57 mmol/g for MC and 2.80 mmol/g for MNAC. In the case of the alkaline groups, a completely opposite relationship was observed. On the surface of MC and MNAC, the highest content of such groups was found: 5.05 for the activated carbon membrane and 5.19 mmol/g for the nitrogen-enriched activated carbon modified membrane, respectively. A slightly lower amount of 4.60 mmol/g was found for the MAC material. In contrast, the least alkaline functional groups were present on the surface of the membrane without the addition of carbon materials and amounted to 1.66 mmol/g. An analysis of the data presented in Table 4 indicated that the addition of carbon adsorbent to the membrane results in an increase in the number of basic groups compared to the membrane without carbon addition. With regard to acidic groups, no discernible trend was apparent. Consequently, further research should be conducted with the objective of resolving this issue.

**Figure 4:**
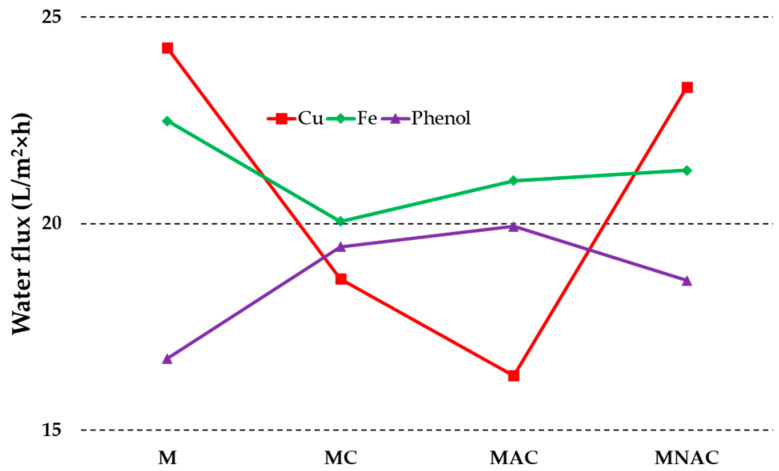
Water flux of the membranes before and after copper, iron and phenol filtrations.

Figure 4 illustrates the flow rates determined for the materials under investigation. The highest flow values were observed for the M membrane, with a value of 24.25 L/m^2^×h prior to filtration of copper ion solutions. A slightly lower value of 23.30 L/m^2^×h was determined for the MNAC membrane, while the flow values for the MC and MAC membranes were 18.66 and 16.32 L/m^2^×h, respectively. The flow values for the materials used to remove iron ions were similar to each other. The value for the M membrane was 22.48 L/m^2^×h, for the MC material 20.05 L/m^2^×h and for the MAC and MNAC materials 21.04 and 21.29 L/m^2^×h, respectively. The highest flow rates for MC and MAC were 19.44 and 19.93 L/m^2^×h, respectively, for the materials used in the phenol filtration processes. M and MNAC membranes exhibited slightly lower values for this parameter, with values of 16.73 L/m^2^×h for M and 18.62 L/m^2^×h for MNAC.

**Figure 5:**
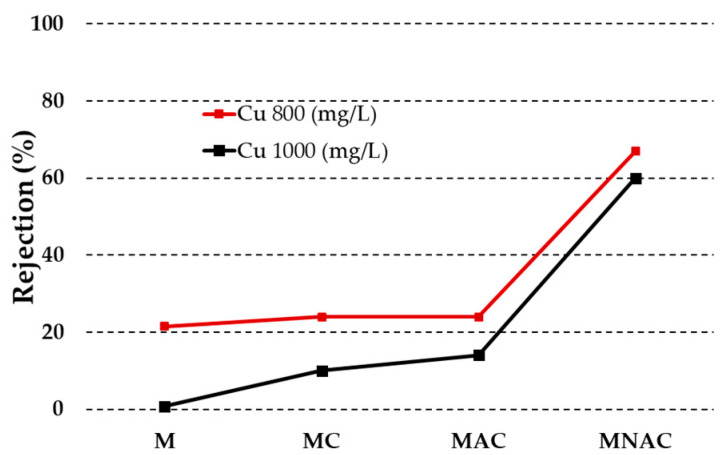
Removal of copper ions by membranes obtained.

Figure 5 illustrates the efficacy of the materials tested in removing copper ions from aqueous solutions. All of the materials exhibited enhanced efficiency in removing copper ions from solutions with an initial concentration of 800 mg/L. The results indicate that copper ions, regardless of the initial solution concentration, are most effectively removed by the urea-impregnated carbon membrane. It is possible that the observed trend is related to the fact that NAC coal was characterised by the best developed specific surface area and porous structure. The material was found to be highly effective in removing copper ions from aqueous solutions, with 67% of the ions removed from a solution with an initial concentration of 800 mg/L and 60% from a solution with a higher concentration. In contrast, the use of the MAC material resulted in the removal of only 24% of the ions from a solution with an initial concentration of 800 mg/L and 14% from a solution with an initial concentration of 1000 mg/L. In contrast, the use of the MC membrane allowed the removal of 24 and 10% of copper ions, respectively. The membrane without added carbon material was the least effective in removing copper ions from aqueous solutions, removing 21% of the ions from a solution with a lower initial concentration and only 1% from a solution with an initial concentration of 1000 mg/L.

The effectiveness of the adsorbents tested for the removal of copper ions can be compared with results already reported in the literature. It should be emphasised that the preparation of the membrane in the form of flat sheets can be considered as a simple process and therefore economical. In addition, the use of carbonaceous adsorbents from waste materials does not significantly increase operating costs. Comparing the results obtained in this article with those reported in [27], where the authors present their findings on the performance of membranes impregnated with polysulfone minerals and clay for Cu(II) ion remediation, it can be concluded that the materials obtained are comparable. Indeed, the authors in [27] used mixing and phase inversion methods to prepare clay-based membranes by mixing bentonite, sepiolite and zeolite in a matrix for the removal of copper ions from aqueous solutions. It was shown that for a final concentration of copper ions equal to 5 mg/L (TMP = 0.5 bar), the following rejections were obtained: 59.2% bentonite/polysulfone, 21.5% sepiolite/polysulfone and 97.0% zeolite/polysulfone. In [28], a membrane with a mixed biochar/polysulfone (PSF) matrix was prepared by incorporating biochar microparticles into the PSF matrix. The biochar was obtained by physical activation of plant waste. It was shown that the rejection was up to 93.7% (Co—12.8 mg/L, TMP = 0.25 bar), which is a much higher value compared to our results.

**Figure 6:**
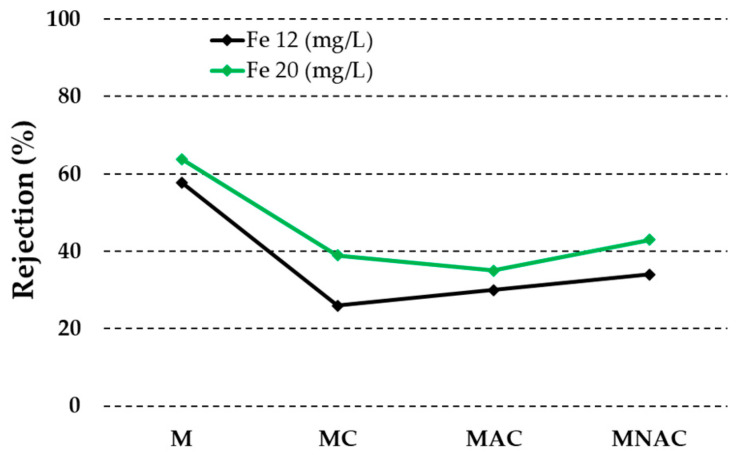
Removal of iron ions by membranes obtained.

A quantitative analysis of the data presented in Figure 6 reveals that all the membranes tested exhibited enhanced efficiency in the removal of iron ions from aqueous solutions with an initial concentration of 20 mg/L. The values collected indicate that the membrane without added carbon material exhibited the highest efficiency in the removal of iron ions. The membrane was found to be highly effective in removing iron ions from aqueous solutions. It was observed that 64% of the iron ions were removed from a solution with an initial concentration of 20 mg/L, while 58% were removed from a solution with a concentration of 12 mg/L. In comparison, the results obtained for the other materials were considerably lower. The MC material demonstrated iron ion removal efficiencies of 26% for the lower concentration solution and 39% for the 20 mg/L solution. The use of MAC material resulted in the removal of 30% of the ions from a solution with an initial concentration of 12 mg/L, while 35% of the ions of this metal were removed with MNAC material. In contrast, the use of MAC material removed 34% of the ions from a solution with an initial concentration of 20 mg/L, while 43% of the ions of this metal were removed with MNAC material.

The scientific literature provides evidence that the removal of ions, including iron, by membranes is of interest to many research groups. For example, ref. [29] described the synthesis of graphene oxide and its incorporation into membranes made from recycled PET bottles. The study showed that the rejection rate was 60% (at an initial concentration of C_0_ = 0.3 mg/L and TMP pressure = 0.15 bar). It is notable that the incorporation of graphene oxide into PET bottle waste materials as a raw material for the production of membranes for wastewater treatment, as demonstrated in our work, allows the waste to be utilized for the fabrication of effective adsorbents for liquid phase contaminants. In turn, a ceramic membrane containing activated carbon derived from empty oil palm fruit bunches showed an iron ion removal efficiency of over 92% (C_0_ = 1.38 mg/L, TMP = 2.0 bar) [30]. Polymer membranes based on polyethersulfone, into which PVP (polyvinylpyrrolidone, a pore-generating agent) was also introduced, exhibited a rejection efficiency of over 90% (C_0_ = 5–20 mg/L, TMP = 3.0 bar) [31].

**Figure 7:**
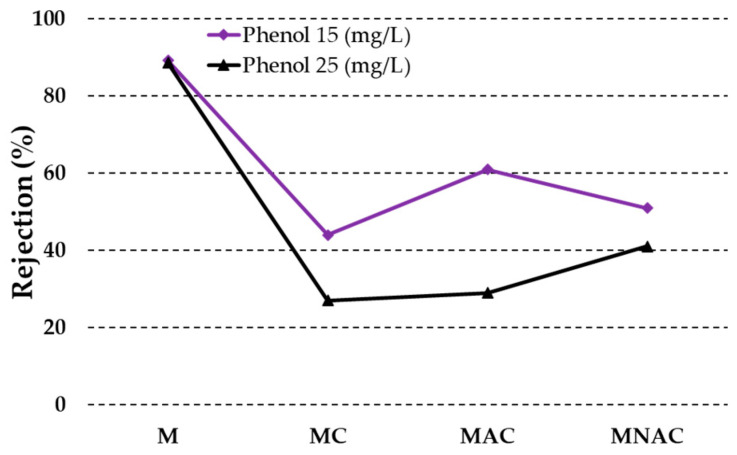
Removal of phenol by membranes obtained.

As with the removal of copper ions, the membranes tested demonstrated enhanced efficiency in the removal of phenol molecules from solutions with lower initial concentrations (Figure 7). Regardless of the initial concentration of the solution, the membrane without added carbon materials exhibited the highest efficacy (89%). The other materials exhibited significantly lower efficiency in the removal of phenol from aqueous solutions. The use of MC allowed for the removal of 44% of phenol from a solution with an initial concentration of 15 mg/L and 27% from a solution with an initial concentration of 25 mg/L. In contrast, the use of MAC resulted in the removal of 61% and 29% of phenol molecules, respectively. In contrast, MNAC permitted the removal of 51 and 41% of phenol molecules from solutions with initial concentrations of 15 and 25 mg/L, respectively.

Given the adverse effects of phenol on human health, numerous research groups are engaged in investigating methods for its removal from wastewater. In [32], researchers employed a composite membrane prepared by coating a mixture of choline chloride and cellulose acetate based on fly ash ceramic substrate for phenol removal. Their findings demonstrated that phenol rejection increased from 56 to 93% with a concomitant increase in the quantity of choline chloride in cellulose acetate. Our previous research [33] indicates that the use of polymeric membranes based on polyethersulfone (PES) modified by the addition of varying quantities of a pore-forming agent (PVP) allows for a significantly higher removal of phenol from the liquid phase (rejection near 100%) compared to the results presented in this paper. Consequently, it can be postulated that the introduction of carbonaceous adsorbents does not have a positive effect on the results obtained when removing this type of contamination.

**Figure 8:**
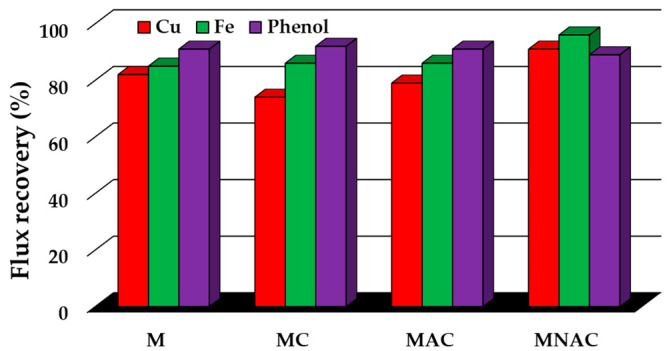
Flux recovery percentage of investigated membranes after filtrations.

Figure 8 illustrates the mean values of the renewal rate of the individual membranes following filtration of the solutions tested. The results demonstrate that the highest values for this parameter were observed for the MNAC material, with values of 91% following the filtration of copper ion solutions, 96% following filtration of iron ions and 89% following filtration of phenol. The remaining materials exhibited slightly lower values. The FRR values for M were 82, 85 and 91% after filtration of copper, iron and phenol solutions, respectively. In contrast, the FRR values for MC were 74% after copper filtration, 86% after iron filtration and 92% after phenol filtration. Finally, the FRR values for MAC were 79, 86 and 91% after filtration of copper, iron and phenol solutions, respectively. From the graph obtained, it can be seen that in most cases (for M, MC and MAC materials), the FRR values increase depending on the type of filtered solution. The lowest values were obtained after filtration of copper solutions, followed by increasing values after filtration of ferrous ion solutions, while the highest values were observed after phenol removal processes from aqueous solutions.

Table 5 presents the mean values of the individual resistances determined for the materials obtained following the filtration of copper ion solutions. The data indicate that the highest resistances (both for the membranes, pores and the resulting filter cake) are those of the MAC material, with a total filtration resistance of 25.26 × 10^13^ L/m^2^×h. The MC membrane exhibited slightly lower values, with an R_t_ of 22.66 × 10^13^ L/m^2^×h. In contrast, the M and MNAC materials exhibited lower resistance values. The total filtration resistances for these membranes are 16.27 × 10^13^ L/m^2^×h for M and 14.32 × 10^13^ L/m^2^×h for MNAC, respectively.

Table 6 presents the average resistance values determined following the iron ion filtration processes. The highest values for individual resistances and total filtration resistance were observed for the MC membrane, with R_t_ equal to 18.30 × 10^13^ L/m^2^×h. Slightly lower values were found for MAC, with the total filtration resistance equalling 17.50 × 10^13^ L/m^2^×h. The resistance values for M and MNAC are slightly lower than those determined for MC and MAC membranes. The total filtration resistance for M is 15.83 × 10^13^ L/m^2^×h and 15.51 × 10^13^ L/m^2^×h for MNAC.

The average resistance values determined following filtration of the phenol solutions are presented in Table 7. The values obtained indicate that all materials exhibit comparable resistance values. The Rt value for M is 17.19 × 10^13^ L/m^2^×h, while for MC it is equal to 17.22 × 10^13^ L/m^2^×h. In contrast, the total filtration resistance value for MAC is 17.69 × 10^13^ L/m^2^×h, while that for MNAC is 18.61 × 10^13^ L/m^2^×h.

## 4. Conclusions

The carbonaceous adsorbents obtained from the extraction residues of the fungus *Inonotus obliquus* exhibited a specific surface area of 205 to 644 m^2^/g and contained both acidic and basic functional groups on their surface, with their aqueous solutions displaying an alkaline character. This study demonstrated that urea impregnation produced an adsorbent with the highest carbon content.

This study demonstrated that the incorporation of char, derived from the carbonisation of the extraction residue of the fungus *Inonotus obliguus*, into the membrane resulted in the highest porosity values and equilibrium water content. Conversely, the membrane enriched with AC material exhibited the highest surface hydrophilicity. Furthermore, the addition of activated carbon, obtained through physical activation of char, led to an increase in the content of acidic oxygen groups on the surface of the membrane under study. In contrast, membranes based on char and nitrogen-enriched activated carbon have a significantly higher number of basic groups on their surface than acidic groups. A membrane with carbon material containing urea in its structure shows the highest efficiency in removing copper ions. In contrast, a membrane without added carbon materials shows the highest efficiency in removing phenol and iron ions from aqueous solutions. Urea-impregnated activated carbon has a positive effect on the renewal rate of membranes. The research findings indicate that the addition of carbonaceous material has an inconclusive effect on the removal efficiency of the pollutants tested. The carbon materials obtained were characterised by a poorly developed specific surface area and porous structure, which suggests that further research should be directed towards the use of carbon adsorbents obtained by chemical activation of waste materials. This method allows a much more effective development of the surface area of carbonaceous material. The next step is to confirm the usefulness of the research carried out. This can be achieved by conducting stability and reusability studies of the obtained materials on model systems and real wastewater. Additionally, a cost analysis of the membranes produced should be carried out to enable a comparison of their production costs with those of commercial products currently available on the market.

## Figures and Tables

**Table 1:** Elemental analysis (wt. %) of the carbon materials.

Carbon Materials	C^daf^	H^daf^	N^daf^	S^daf^	O^daf,1^	Ash
C	69.2	2.1	2.9	0.2	25.7	12.1
AC	74.6	2.5	3.0	0.1	19.8	14.3
NAC	86.9	2.7	4.7	0.1	5.6	7.8

^daf^—dry-ash-free basis; ^1^—determined by difference, method error ≤ 0.3%.

**Table 2:** Textural parameters of the carbon materials.

Carbon Materials	Iodine Number (mg/g)	S_BET_ (m^2^/g)	Total Pore Volume (cm^3^/g)	Micropore Volume (cm^3^/g)	Average Pore Diameter (nm)
C	205	125	0.38	0.04	7.32
AC	356	749	0.52	0.39	4.22
NAC	644	888	0.60	0.44	4.15

S_BET_—surface area

**Table 3:** Porosity (ε), equilibrium water content (EWC) and contact angle of investigated membranes (standard deviation n = 10).

Membrane	ε (%)	EWC (%)	Contact Angle °
M	50.31 ± 2.61	76.74 ± 4.61	62.80 ± 1.47
MC	68.60 ± 3.79	88.21 ± 5.28	60.08 ± 4.09
MAC	38.01 ± 1.14	80.13 ± 4.17	57.12 ± 2.97
MNAC	62.71 ± 3.19	85.62 ± 4.88	58.24 ± 3.57

**Table 4:** Acidic and basic properties of investigated adsorbents (mmol/g).

Membrane	Acidic Groups	Basic Groups	Total Content of Oxygen Groups
M	3.69 ± 0.23	1.66 ± 0.11	5.35 ± 0.34
MC	2.57 ± 0.17	5.05 ± 0.32	7.62 ± 0.37
MAC	5.19 ± 0.32	4.60 ± 0.28	9.79 ± 0.45
MNAC	2.80 ± 0.21	5.19 ± 0.32	7.99 ± 0.39

**Table 5:** Filtration resistance of membranes after filtrations of copper ion solutions (L/m^2^×h) (standard deviation n = 10).

Membrane	R_m_ (×10^13^)	R_p_ (×10^13^)	R_c_ (×10^13^)	R_t_ (×10^13^)
M	4.65 ± 0.19	5.82 ± 0.34	5.80 ± 0.31	16.27 ± 1.05
MC	5.79 ± 0.23	7.80 ± 0.57	9.07 ± 0.88	22.66 ± 1.45
MAC	6.64 ± 0.24	8.42 ± 0.68	10.20 ± 0.93	25.26 ± 2.99
MNAC	4.65 ± 0.20	5.15 ± 0.43	4.52 ± 0.42	14.32 ± 0.99

**Table 6:** Filtration resistance of membranes after filtrations of iron ion solutions (L/m^2^×h) (standard deviation n = 10).

Membrane	R_m_ (×10^13^)	R_p_ (×10^13^)	R_c_ (×10^13^)	R_t_ (×10^13^)
M	4.87 ± 0.18	5.71 ± 0.53	5.25 ± 0.52	15.83 ± 1.59
MC	5.49 ± 0.24	6.36 ± 0.77	6.45 ± 0.65	18.30 ± 1.78
MAC	5.14 ± 0.21	5.98 ± 0.49	6.38 ± 0.63	17.50 ± 1.68
MNAC	5.09 ± 0.24	5.34 ± 0.47	5.08 ± 0.49	15.51 ± 1.56

**Table 7:** Filtration resistance of membranes after filtrations of phenol solutions (L/m^2^×h) (standard deviation n = 10).

Membrane	R_m_ (×10^13^)	R_p_ (×10^13^)	R_c_ (×10^13^)	R_t_ (×10^13^)
M	6.59 ± 0.88	7.19 ± 0.94	6.41 ± 0.82	17.19 ± 1.48
MC	5.58 ± 0.79	6.07 ± 0.84	5.57 ± 0.57	17.22 ± 1.47
MAC	5.62 ± 0.81	6.12 ± 0.83	5.95 ± 0.59	17.69 ± 1.54
MNAC	5.80 ± 0.84	6.54 ± 0.87	6.27 ± 0.63	18.61 ± 1.59

## Data Availability

The original contributions presented in the study are included in the article, further inquiries can be directed to the corresponding author.
